# Vulnerability Mining Method for the Modbus TCP Using an Anti-Sample Fuzzer

**DOI:** 10.3390/s20072040

**Published:** 2020-04-05

**Authors:** Yingxu Lai, Huijuan Gao, Jing Liu

**Affiliations:** College of Computer Science, Faculty of Information Technology, Beijing University of Technology, Beijing 100124, China; laiyingxu@bjut.edu.cn (Y.L.); g_h_j1205@163.com (H.G.)

**Keywords:** industrial control system, Modbus TCP, probability distribution, recurrent neural network, vulnerability mining

## Abstract

Vulnerability mining technology is used for protecting the security of industrial control systems and their network protocols. Traditionally, vulnerability mining methods have the shortcomings of poor vulnerability mining ability and low reception rate. In this study, a test case generation model for vulnerability mining of the Modbus TCP based on an anti-sample algorithm is proposed. Firstly, a recurrent neural network is trained to learn the semantics of the protocol data unit. The softmax function is used to express the probability distribution of data values. Next, the random variable threshold and the maximum probability are compared in the algorithm to determine whether to replace the current data value with the minimum probability data value. Finally, the Modbus application protocol (MBAP) header is completed according to the protocol specification. Experiments using the anti-sample fuzzer show that it not only improves the reception rate of test cases and the ability to exploit vulnerabilities, but also detects vulnerabilities of industrial control protocols more quickly.

## 1. Introduction

In the era of “Internet +”, the impact of AI, IOT, 5G, cloud computing, and other technologies on industrial activities has become increasingly significant [1]. These technologies have greatly improved productivity and have expanded creativity in the use of industrial control systems (ICS) [2,3,4], but these benefits also bring additional network security risks. For example, there have been a number of security vulnerabilities associated with the MicroLogix 1400 series programmable logic controller (PLC) developed by Rockwell Automation [5], resulting in denial of service attacks, equipment configuration tampering, and other security breaches. In another cybersecurity incident, the website of the Ministry of Energy and Industry of Ukraine was attacked by hackers [6], encrypting the files on the host and causing the website to become inaccessible. These attacks demonstrate the need to protect ICS from network attacks and to maintain their stability [7].

**Figure 1:**
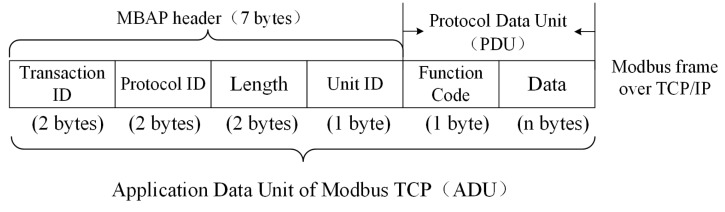
Structure of Modbus TCP. MBAP, Modbus application protocol.

The ICS is composed of three parts: the management layer, the PLC control layer, and the sensing device layer. The industrial control network protocol is a communication protocol between the management layer and the PLC control layer. In the management layer, the operator gives control instructions in the form of a network message. The PLC receives the instruction from the management, updates the PLC data according to the internal control algorithm, and monitors the running status of the sensors in real time. The sensing device layer includes a variety of sensors, which are used to collect data from the industrial site and transmit them to the PLC. In the environment of water tower water level ICS, sensors include water level sensors and valve sensors. The Modbus TCP [8,9,10] is the most widely used industrial control network protocol, which uses a master/slave configuration protocol to communicate requests and responses between equipment. As shown in Figure 1, the application data unit (ADU) of Modbus TCP consists of two parts: the Modbus application protocol (MBAP) header and the protocol data unit (PDU). In the MBAP header, Transaction ID is the identifier of the request and response transaction. Protocol ID is used to identify the Modbus TCP. Unit ID is the identifier of slave equipment in the same network branch, and Length is the byte length sum of Unit ID, Function Code, and Data in PDU. Here, Function Code is the function identifier for a control instruction, and Data is the data information related to the control instruction.

The Modbus TCP itself has a set of abnormality judgment rules. Most of the abnormal data packets can be detected, and when an abnormal data packet is sent to the ICS, it returns an abnormal code [11], which indicates the cause of the error. The Modbus TCP abnormal codes and abnormal descriptions are shown in Table 1.

The basic idea of vulnerability mining is to find security vulnerabilities before system implementation by quickly and reliably generating effective test cases, checking the security to prevent the system from being attacked. Therefore, how to construct a large number of effective test cases quickly has become one of the hot issues in implementing vulnerability mining technology.

The structure of the paper is as follows. Section 1 introduces the research background of ICS and the structure of the Modbus TCP. Section 2 details the related work. In Section 3, the vulnerability method of the Modbus TCP is discussed. Section 4 describes the test case generation model of the industrial control network protocol. Section 5 completes the experiment and analyzes the results. Finally, the conclusion and future research perspective are given in Section 6.

## 2. Related Work

At present, the commonly used network protocol vulnerability mining technology includes manual code audit, fuzzing test, static analysis testing, dynamic analysis testing, and bindiff testing [12]. Among them, fuzzing [13,14] is widely used in the vulnerability mining of the industrial control network protocol, which finds the vulnerability by providing unexpected test cases to the target system and monitoring abnormal results. 

The origins of fuzzing can be traced back to 1989. Professor Barton Miller input random characters into seven types of UNIX variant applications to observe whether the physical program hangs or crashes. As a result, 25–33% of the physical program is abnormal [15]. Gradually, fuzzing test frameworks have been proposed, such as Spike, Peach, and Sulley [16,17]. In recent years, artificial intelligence has gradually entered the field of the industrial Internet. Hu [18] used a generative adversarial network to train real protocol messages, learn protocol semantics, and then generate false, but trusted messages. Finally, he found some errors in the Modbus TCP simulator. Li [19] introduced a vulnerability mining method using Wasserstein generative adversarial networks to generate fuzzing test data, which utilized the Modbus TCP and Ethernet as test targets. The model can be trained within a short time, which is computationally light and practical.

However, the existing vulnerability mining methods generally have some problems of low reception rate and poor vulnerability mining ability. In this case, some vulnerabilities in the ICS may not be discovered. Thus, the security of ICS will have significant hidden dangers, and the safety of factories and countries will also face huge threats. Therefore, it is necessary to improve the reception rate of vulnerability mining methods and enhance their vulnerability mining abilities. 

In order to improve the performance of the test case generation method, we propose an anti-sample algorithm to generate test cases automatically for vulnerability mining of the Modbus TCP. In this paper, our idea is that frequently used test cases have been tested many times, and the probability of finding a new vulnerability is very low; however, rarely used test cases have better vulnerability mining capabilities. According to these ideas, we propose an anti-sample algorithm. A recurrent neural network (RNN) [20,21] is trained to learn the meaning of the Modbus TCP and then outputs the probability matrix of the protocol field value. The field value with the maximum probability in the matrix is the value most frequently used, while the data value with the minimum probability is the value that is least frequently used. Finally, an anti-sample is given, which is composed of protocol field values that are rarely used. In the experimental part of this paper, the effectiveness of our anti-sample algorithm is verified.

## 3. Vulnerability Mining Method for Modbus TCP

In Modbus TCP, the MBAP message header is only the header identification of the protocol, while the Data field contains register fields of operation objects in the industrial control equipment. The operation objects of Modbus TCP include the switch input register, switch output register, analog input register, and analog output register. Each register has its own specific value. For example, the register at address 0x002B stores the humidity value of the industrial control equipment, while the register at address 0x002C stores the dew point value.

### 3.1. Probability Distribution Based on RNN

RNN is a type of deep learning model for sequence data, which can thoroughly understand the dependencies on the regular pattern of the register value in the ICS. For instance, Modbus TCP determines that the Protocol ID is constant 0x00, and the sum of byte length of Unit ID, Function Code, and Data determines the specific value of Length.

**Figure 2:**
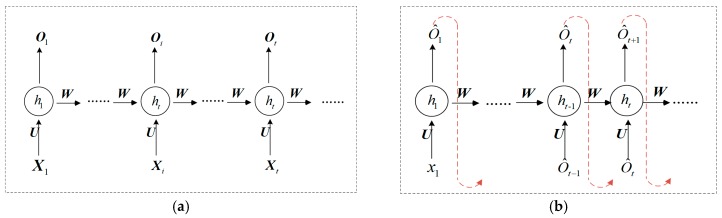
RNN expansion model based on time series: (**a**) the learning stage of the RNN model; (**b**) the generation stage of the RNN model.

The process of generating a test case by RNN is divided into two parts: learning stage and generation stage. It is assumed that there are n messages in the training dataset, and the length of each protocol message is d. The $i^{\mathrm{th}}$ message is represented as $X_{i}=\{x_{1},\ldots ,x_{i},\ldots ,x_{d}\}$, and $x_{i}$ represents the $i^{\mathrm{th}}$ field in the Modbus TCP message. The process of the learning message is shown in Figure 2a, and the input of RNN is $\left\{X_{1},X_{2},\ldots ,X_{i},\ldots ,X_{n}\right\}$. To ensure that the RNN model can learn the semantics in Modbus TCPs, each input value of RNN corresponds to an output value, and the output of RNN is $\left\{O_{1},O_{2},\ldots ,O_{i},\ldots ,O_{n}\right\}$.

The process of the generation stage is shown in Figure 2b. The RNN model is trained, and the model weight parameters remain unchanged. The input value is $x_{1}$, and each step has an output value. ${\hat{O}}_{t}$ represents the output value at time t, based on the generated message sequence $\left\{{\hat{O}}_{1},\ldots ,{\hat{O}}_{t-1}\right\}$ and the current input message data $\left\{{\hat{O}}_{t-1}\right\}$, which is also taken as the input value at time t+1. That is, the RNN generates the conditional probability distribution $P({\hat{O}}_{t}|x_{1},{\hat{O}}_{1},\ldots ,{\hat{O}}_{i},\ldots ,{\hat{O}}_{t-1}\}$ [22] of the output value ${\hat{O}}_{t}$ at the current time t by learning a protocol message sequence $\left\{{\hat{O}}_{1},\ldots ,{\hat{O}}_{t-1}\right\}$.

**Figure 3:**
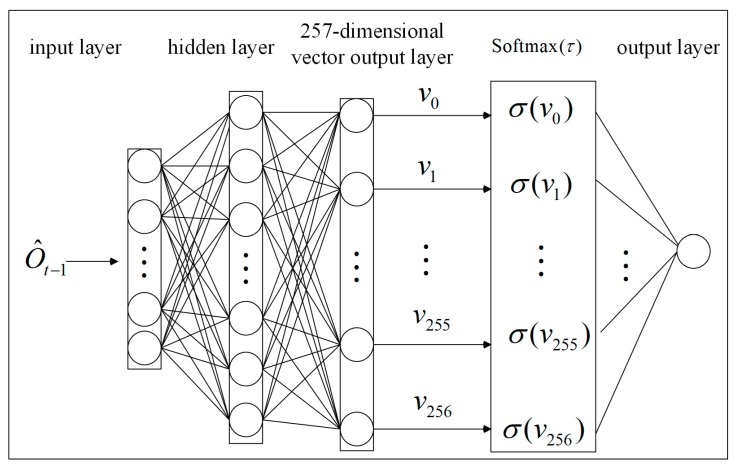
Calculation process of the softmax function.

The softmax function [23,24,25] not only expresses the probability distribution of discrete random variables, but also preserves the gradient information of a neural network. The parameter τ adjusts the smoothness of the probability distribution to change the diversity of the random variable. Therefore, we use a softmax function as the activation function of the last layer of the RNN. Each hexadecimal data value is converted into a decimal integer, and the data value 256 is used as the end flag of a message. As shown in Figure 3, for input data $\left\{{\hat{O}}_{t-1}\right\}$ at time t, the RNN outputs the 257-dimensional vector V, given by Equation (1).

(1) $$V=(v_{0},v_{1},\cdots ,v_{i},\cdots ,v_{255},v_{256})$$

When the softmax function is used to calculate the probability of $v_{i}$, the parameter τ is added (as expressed in Equation (2)) to adjust the probability interval, where the value interval of τ is in [0,∞) [26]. As τ approaches positive infinity, the probability difference is small and tends to be evenly distributed. However, as τ approaches zero, the probability distribution is close to the one-hot encoding form, and the problem of gradient disappearance becomes more serious. The so-called one-hot encoding [27] treats each value of discrete characteristics as a state, and only one state is active at any time.

(2) $$\sigma (v_{i}^{})=P_{v_{j}}=\frac{\exp(v_{i}^{}/\tau )}{\sum_{j=0}^{n}\exp(v_{j}^{}/\tau )}$$

The output probability $P_{v_{i}}$ (given by Equation (3)) of the RNN for each data value $v_{i}$ in vector V is calculated using Equation (2). The higher the probability, the more accurate the semantic learning and the higher the probability of the output value for the target. All probabilities form the output probability distribution matrix O at time t, which describes the probability distribution rule of data values, as expressed as Equation (4).

(3) $$0\leq P_{v_{i}}=P({\hat{O}}_{t}=v_{i})=P_{v_{i}}({\hat{O}}_{t}|\{x_{1},{\hat{O}}_{1},\ldots ,{\hat{O}}_{t-1}\})\leq 1$$

(4) $$O=(P_{0},P_{1},\cdots P_{255},p_{256})$$

### 3.2. Test Case Generation Strategy of Modbus TCP Based on the Anti-sample

Suppose that all possible output results of output value ${\hat{O}}_{t}$ at time *t* constitute sample space $\Omega^{t}$ (as given by Equation (5)), where $\omega_{i}^{t}$ is a basic event [28]. When the basic event $\omega_{i}^{t}$ occurs, the output data value is ${\hat{O}}_{t,i}$, as shown in Equation (6). $P({\hat{O}}_{t}=f(\omega_{i}^{t}))$ represents the probability that ${\hat{O}}_{t}$ is equal to the data value ${\hat{O}}_{t,i}$.

(5) $$\Omega^{t}=\{\omega_{0}^{t},\cdots ,\omega_{i}^{t},\cdots ,\omega_{256}^{t}\}$$

(6) $${\hat{O}}_{t,i}=f(\omega_{i}^{t})=i$$

The output result of the softmax layer is the probability that the predicted value belongs to 257 data values. Usually, the maximum probability is used to express the highest accuracy of neural network learning message semantics, and the data value corresponding to the maximum probability is used as the predicted data value of the input message. 

**Figure 4:**
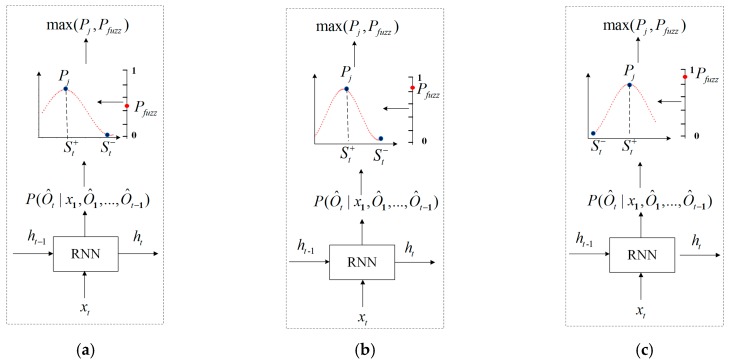
Anti-sample generation strategy: (**a**) $P_{j}>P_{fuzz};$ (**b**) $P_{j}=P_{fuzz};$ (**c**) $P_{j}<P_{fuzz}$.

The anti-sample algorithm in this paper uses the probability distribution to represent the rules of data values in registers of the industrial control equipment and uses the data value with the lowest probability as the output value to generate test cases, thereby mining the vulnerability of the protocol. As shown in Figure 4, the process of generating a test case based on time series is as follows: Firstly, according to the current input data and the hidden information of the learned message, RNN uses a softmax layer to output the probability distribution of the current time. The softmax layer expresses the accuracy of the semantics of the learned message with the probability. Then, for each probability matrix generated by RNN, the algorithm generates a random variable threshold, which is not fixed at each time. Finally, the maximum probability is compared with the variable threshold. According to the comparative result, we decide whether the data value is generated at the current time in accordance with the normal message semantics, thus increasing the possibility of an abnormal occurrence in the industrial control system.

Because the probability values in the probability distribution are between zero and one, the random variable threshold is also between zero and one. This paper abbreviates the strategy of generating test cases by the anti-sample algorithm as the generation strategy. The Modbus TCP vulnerability mining fuzzer, constructed using the anti-sample algorithm, is called the anti-sample fuzzer (A-s fuzzer).

In Equations (7)–(9), $P_{j}$ and $P_{i}$ are the maximum probability and the minimum probability of the probability matrix, respectively; ζ is the RNN model; O is the probability distribution matrix of the output ζ; and $P_{fuzz}$ is the random variable threshold.

(7) $$P_{j}=P({\hat{O}}_{t}=f(\omega_{j}^{t}))=\underset{\xi }{\max}(O)$$

(8) $$P_{i}=P({\hat{O}}_{t}=f(\omega_{i}^{t}))=\underset{\xi }{\min}(O)$$

(9) $$P_{fuzz}=\mathrm{random}(0,1)$$

In the anti-sample generation strategy, when $P_{j}$ is greater than or equal to $P_{fuzz}$ (as shown in Figure 4a,b), it indicates that the data values accessed by the register have definite regularity and often appear in a certain position. Therefore, we act in a diametrically opposite way; at the current time, the data value of the minimum probability is used to replace the data value of the maximum probability to generate a message. Such test cases are typically rarely used in the communication between the industrial control tester and the industrial control equipment, but they may cause serious protocol vulnerabilities. For example, in industrial control equipment, access the register of address 0x002B to read the humidity value, which is often the data value $S_{t}^{+}$(as shown in Equation (10)). If $S_{t}^{-}$ (as shown in Equation (11)) is selected as the humidity value at this time, such test cases are rarely used in vulnerability mining, but may cause the vulnerability of the protocol.

When $P_{j}$ is less than $P_{fuzz}$ (as shown in Figure 4c), it indicates that there is no definite regularity in the data value accessed by the register, and it is not necessary to change the access mode of the register. For example, in industrial control equipment, access the register of address 0x002C to read the dew point value. In rare cases, the dew point value is the data value $S_{t}^{+}$, and selecting the data value $S_{t}^{+}$ as the dew point value for vulnerability mining may cause abnormal or no response of industrial control equipment.

(10) $$S_{t}^{+}=f(\omega_{j}^{t})=\underset{\xi }{\arg\;\max}(O)$$

(11) $$S_{t}^{-}=f(\omega_{i}^{t})=\underset{\xi }{\arg\;\min}(O)$$

The fields in the protocol message are closely related such that a complete test case depends on both MBAP and PDU. Therefore, it is necessary to add the message header part of MBAP based on the message generated by the generation strategy and then construct a normal test case to monitor the state of the tested object. The PDU component of the Modbus TCP message is generated according to the generation strategy of the model, while the MBAP message header needs to be randomly generated according to the Modbus TCP. In this way, the MBAP header and PDU are spliced together to form a complete test case for the industrial control protocol vulnerability mining system.

## 4. Test Case Generation Model of the Industrial Control Network Protocol

**Figure 5:**
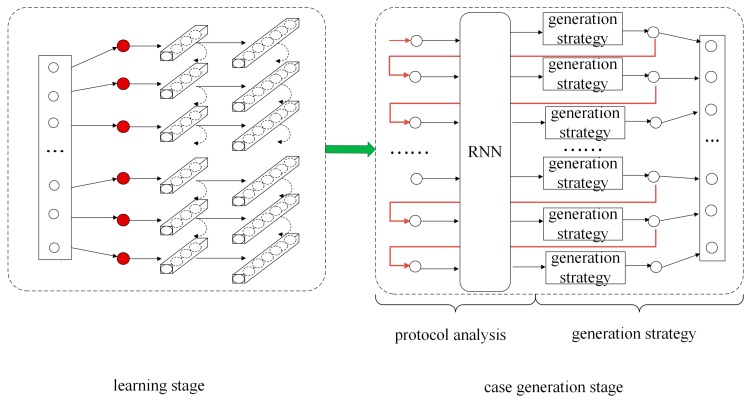
Generation model structure diagram of the industrial control network protocol.

In this section, we first present an overview of the generation model and then elaborate on the stages. The overall architecture is illustrated in Figure 5.

### 4.1. Overview

The structure of the model for test case generation in this paper consists of two parts: learning stage and case generation stage. The learning stage is used to learn the message of the training dataset and determine the weight parameters of RNN. The case generation stage is divided into protocol analysis and generation strategy. In protocol analysis, when a new field is input, RNN can determine the probability distribution relationship of the next generated field based on the learning of Modbus TCP message rules. The generation strategy is responsible for changing the information attached to its probabilistic relationship to generate the next protocol field. The changed field will be the input information for the RNN at the next time. 

### 4.2. Learning Stage

**Figure 6:**
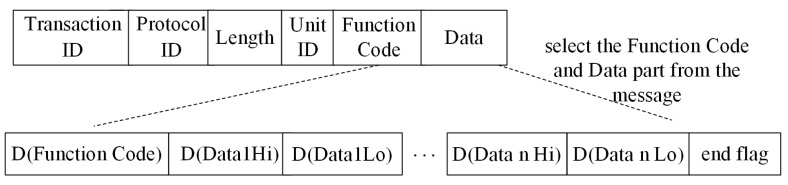
The construction of learning messages and the conversion process of data values in Modbus TCP.

For each Modbus TCP message, the Function Code and Data of each protocol are selected. The two byte message fields are divided into high byte and low byte according to the high byte data and the low byte data, and the hexadecimal data values in each byte are converted to decimal data values. As shown in Figure 6, *D* represents a function that converts hex to decimal. Because the hexadecimal data range of a byte is 0x00–0xFF, after it is converted to decimal, the value range of each data value is 0–255, and all are integers.

Suppose that there are num Modbus TCP messages; the sum of all message lengths is n. First, the message is extracted according to the format of Figure 6, and the end flag’s data value 256 is added at the end of each message. That is, the value range of each data value is an integer in the range of 0–256. Thus, the sum of the learned message length is n−num⋅7+num⋅1, where 7 is the length of the header byte of the MBAP message and 1 is the length of the increased end flag data value. Because the input data matrix of the RNN model is the same size, we concatenate all the messages to form a large matrix S during training, and the order of the fields in the messages remains unchanged. Finally, starting from the first data value of S, each interval of m data values constitutes a message input during model learning, and starting from the second data value, each interval of m data values constitutes a message output during model training. Therefore, the $i^{\mathrm{th}}$ input message of the model is $\left\{S_{i},\ldots ,S_{i+m}\right\}$, and the $i^{\mathrm{th}}$ output message is $\left\{S_{i+1},\ldots ,S_{i+m+1}\right\}$, where the length of each message is m, and there are ⌊(n−num⋅7+num⋅1−1)/m⌋ messages in total. The subscript i represents the $i^{\mathrm{th}}$ message. 

Assume that the matrix composed of input messages in the Modbus TCP training dataset is X, as shown in Equations (12) and (13), where $X_{i}$ represents the $i^{\mathrm{th}}$ input message. The matrix composed of the expected output message is Y, as shown in Equations (14) and (15). $Y_{i}$ represents the $i^{\mathrm{th}}$ expected output message.

(12) $$X=\left[X_{0},X_{1},\ldots ,X_{i},\ldots ,X_{\left\lfloor num-1/m\right\rfloor }\right]$$

(13) $$X_{i}=\{S_{i},S_{i+1},\ldots ,S_{i+m-1}\}$$

(14) $$Y=\left[Y_{0},Y_{1},\ldots ,Y_{i},\ldots ,Y_{\left\lfloor num-1/m\right\rfloor }\right]$$

(15) $$Y_{i}=\{S_{i+1},S_{i+2},\ldots ,S_{i+1+m-1}\}$$

In the learning phase, the two-layer RNN is adopted to design the distribution rule for learning the protocol data value. Based on the time series expansion, the input message is X, the expected output message is Y, and the input message at each time is $X_{i}^{}$.

In the learning stage of this paper, the probability distribution θ of the real output ${\hat{Y}}_{i}$ is denoted as ${\hat{Y}}_{\left[i\right]}$, and the expected output $Y_{i}$ is converted to the polynomial distribution vector of one-hot encoding as $Y_{\left[i\right]}$. Next, a value describing the distance between them is given, and the distance sum of all messages is accumulated as the loss function learned [29], which is given by:

(16) $$loss(Y,\hat{Y})=-\frac{\sum_{i=0}^{\left\lfloor \left(n-num\cdot 7+num\cdot 1-1\right)/m\right\rfloor }Y_{\left[i\right]}\times \log{\hat{Y}}_{\left[i\right]}^{}}{\left\lfloor \left(n-num\cdot 7+num\cdot 1-1\right)/m\right\rfloor }$$

### 4.3. Case Generation Stage

The case generation stage includes the protocol analysis and case generation strategy, which is the main part of the generation model.

#### 4.3.1. Protocol Analysis

**Figure 7:**
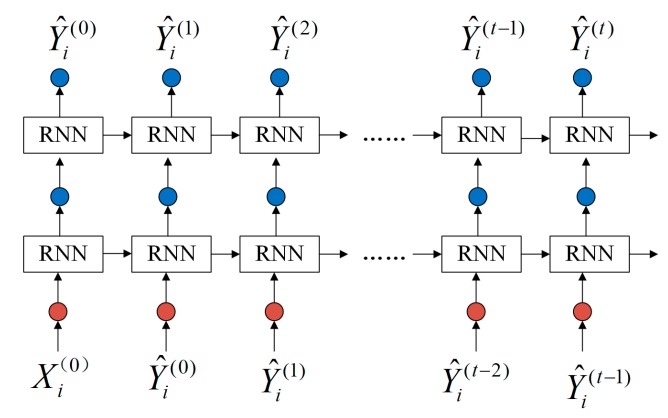
Input-output of the protocol semantic analysis part in the generation phase.

After the learning stage, the internal weight network parameters of the RNN model are determined. When generating the $i^{\mathrm{th}}$ test case, the superscript (t) represents the data value of the $i^{\mathrm{th}}$ test case at time t. In the protocol analysis, the softmax function outputs the probability distribution relation matrix of each time. The RNN model input and output data values change as shown in Figure 7. The initial input to the model is $X_{i}^{\left(0\right)}$, and at each time, the model outputs a data value; this data value is then used as the input for the next time.

Each time has a probability output matrix. There are 257 data values stored in the matrix, and their probability $\theta_{j}$ is the output value; the sum of the total probabilities is equal to one. These probabilities are expressed by Equations (17)–(19), where ξ represents a two-layer RNN model. The actual output probability matrix θ of ξ satisfies Equation (17); the data value corresponding to the maximum probability in the matrix is ${{\hat{Y}}_{i}^{\left(t\right)}}_{}^{+}$, which satisfies Equation (18); and the data value corresponding to the minimum probability in the matrix is ${{\hat{Y}}_{i}^{\left(t\right)}}_{}^{-}$, which satisfies Equation (19).

(17) $$\theta =(\theta_{0},\theta_{1},\cdots ,\theta_{256})={\left(\begin{array}{c} P({\hat{Y}}_{i}^{\left(t\right)}=0|\{X_{i}^{\left(0\right)},\;{\hat{Y}}_{i}^{\left(0\right)},\;{\hat{Y}}_{i}^{\left(1\right)},\;\ldots ,\;{\hat{Y}}_{i}^{\left(t-1\right)}\})\\ P({\hat{Y}}_{i}^{\left(t\right)}=1|\{X_{i}^{\left(0\right)},\;{\hat{Y}}_{i}^{\left(0\right)},\;{\hat{Y}}_{i}^{\left(1\right)},\;\ldots ,\;{\hat{Y}}_{i}^{\left(t-1\right)}\}))\\ \vdots \\ P({\hat{Y}}_{i}^{\left(t\right)}=256|\{X_{i}^{\left(0\right)},\;{\hat{Y}}_{i}^{\left(0\right)},\;{\hat{Y}}_{i}^{\left(1\right)},\;\ldots ,\;{\hat{Y}}_{i}^{\left(t-1\right)}\})) \end{array}\right)}^{T}$$

(18) $${{\hat{Y}}_{i}^{\left(t\right)}}_{}^{+}=\underset{\xi }{\arg\;\max}(\theta )$$

(19) $${{\hat{Y}}_{i}^{\left(t\right)}}_{}^{-}=\underset{\xi }{\arg\;\min}(\theta )$$

#### 4.3.2. Generation Strategy

When the data value ${\hat{Y}}_{i}{}^{\left(j\right)}(j\in [0,t-1])$ of each time is generated based on the anti-sample generation strategy, the specific steps are as follows:

(1) The probability distribution relation matrix θ of the protocol analysis part is output according to the learned semantics and the current input $\left(X_{i}^{\left(0\right)}\;or{\hat{Y}}_{i}^{\left(j-1\right)}\right)$.
(2) There is a random variable threshold $P_{fuzz}$ when each probability matrix is generated. The generated strategy model outputs the maximum probability $\theta_{j}$ in the matrix (given by Equation (20)). If $\theta_{j}$ is greater than or equal to $P_{fuzz}$, ${{\hat{Y}}_{i}^{\left(t\right)}}_{}^{-}$ is selected as the output value. Otherwise, ${{\hat{Y}}_{i}^{\left(t\right)}}_{}^{+}$ continues to be selected as the output value.
  (20) $$\theta_{j}=\underset{\xi }{\max}(\theta )$$
(3) The data value output by the current time is checked against the end flag data value of 256. If it is not the end flag data value, we repeat Steps (1) and (2); otherwise, end the algorithm.

As shown in Algorithm 1, the input of the anti-sample algorithm is the generated message $\left[{\hat{Y}}_{i}^{\left(0\right)},{\hat{Y}}_{i}^{\left(1\right)},\ldots ,{\hat{Y}}_{i}^{\left(t-1\right)}\right]$, the double-layer RNN model ξ, and the output is the generated PDU message ${\hat{Y}}_{i}$.
**Algorithm 1: Anti-Sample Algorithm**input: ζ←the double-layer RNN model;    $\left[{\hat{Y}}_{i}^{\left(0\right)},{\hat{Y}}_{i}^{\left(1\right)},\ldots ,{\hat{Y}}_{i}^{\left(t-1\right)}\right]$←generated message; output: ${\hat{Y}}_{i}$←the generated PDU message ▽based on the generated message $\left[{\hat{Y}}_{i}^{\left(0\right)},{\hat{Y}}_{i}^{\left(1\right)},\ldots ,{\hat{Y}}_{i}^{\left(t-1\right)}\right]$, model ζ outputs its probability distribution matrix θ. (1)**do**$P_{fuzz}\leftarrow random(0,1)$, $\theta_{j}\leftarrow \underset{\xi }{\max}(\theta )$(2)  ${{\hat{Y}}_{i}^{\left(t\right)}}_{}^{+}\leftarrow \underset{\xi }{\arg\;\max}(\theta )$, ${{\hat{Y}}_{i}^{\left(t\right)}}_{}^{-}\leftarrow \underset{\xi }{\arg\;\min}(\theta )$(3)  **if** $\theta_{j}\geq P_{fuzz}$(4)    **then** ${\hat{Y}}_{i}^{\left(t\right)}\leftarrow {{\hat{Y}}_{i}^{\left(t\right)}}_{}^{-}$(5)    **else** ${\hat{Y}}_{i}^{\left(t\right)}\leftarrow {{\hat{Y}}_{i}^{\left(t\right)}}_{}^{+}$(6)  ${\hat{Y}}_{i}\leftarrow {\hat{Y}}_{i}+{\hat{Y}}_{i}^{\left(t\right)}$(7)  t←t+1(8)**do while**${\hat{Y}}_{i}^{\left(t\right)}!=256$ ▽The end flag stops the loop(9)**end while**

Based on the dependent relationship in Modbus TCP, the Function Code and Data parts of the test cases are constructed, and then, complete test cases are generated according to Algorithm 2. First, Transaction ID, Protocol ID (two bytes), and Unit ID (one byte) are randomly generated. Next, Length is calculated according to the number of bytes of Function Code, Data, and Unit ID, and they are converted into two bytes of hexadecimal values. Finally, all values are spliced in order according to the protocol format, as shown in Algorithm 2.
**Algorithm 2: Complete Test Case Generation Algorithm**input: ${\hat{Y}}_{i}$←generated PDU message output: ${\hat{Y}}_{i}$←complete test case (1)unit←hex(random.Int(0,255)) ▽ decimal to hex(2)$length\leftarrow \mathrm{hex}(\mathrm{len}({\hat{Y}}_{i})+1)$(3)transaction_ID← hex(random.Int(0,65535))(4)protocol_ID←hex(0)(5)${\hat{Y}}_{i}\leftarrow (transaction\_ID,\;protocol\_ID,\;length,\;unit,{\hat{Y}}_{i})$

## 5. Experiment Results

In this study, the Modbus TCP is used as the test target to detect the vulnerability of the Modbus TCP in the industrial control equipment.

### 5.1. Training Dataset Selection

The training dataset selected in this study conformed to the diversity and unique characteristics, which were all from real and simulated industrial sites, including natural gas, power grids, and water levels of water towers. Dataset 1 [30] was the industrial control intrusion detection standard dataset established by the infrastructure protection center of Mississippi State University in 2014. The data source was the network layer data of a natural gas pipeline SCADA control system. It included eight types of attack behaviors. We screened out Modbus TCP packets for normal behavior. Dataset 2 [31] generated representative markup datasets for SCADA networks and was proposed at the 2016 Cyber Security Experimentation and Test (CSET) conference. The datasets were generated in a SCADA sandbox at the network level, where electrical network simulators were used to introduce realism for the physical components. It included packet captures including both malicious and non-malicious Modbus traffic and accompanying CSV files. Dataset 3 was the Modbus TCP message supplemented by intrusion detection system of water tower water level in our laboratory. 

At present, there are relatively few datasets in the field of industrial control. We found these three representative datasets. Dataset 1 included function codes 0x03, 0x08, and 0x10. Dataset 2 included function codes 0x01, 0x02, 0x03, 0x05, 0x06, and 0x10. Dataset 3 included function codes 0x01-0x06, 0x0f, and 0x10. The datasets were complementary to each other, covering all commonly used Modbus TCP function codes, ensuring the diversity of Modbus TCP messages. All dataset information came from real or simulated industrial sites, ensuring data reliability.

### 5.2. Experimental Environment and Parameters of the Model

In the process of vulnerability mining of the Modbus TCP, the test cases generated by the industrial control tester were sent to the industrial control equipment, and the equipment returned the corresponding response message or returned the abnormal status according to the received message. The industrial control tester received the response message and sent new test cases again. Every time a test case was sent, we observed the state of the industrial control equipment to determine whether the test case caused abnormal response of the equipment, so as to find the vulnerability of the protocol. The industrial control equipment would have two abnormal states: When the equipment responded to abnormal message, it indicated that this test case caused a protocol vulnerability. When the equipment did not respond, we sent this test case again and then sent two test cases with a normal response. If the equipment did not respond to this test case and could respond normally to the latter two test cases, it meant that the test case was discarded by the equipment. Otherwise, it meant that the test case caused a protocol vulnerability.

**Figure 8:**
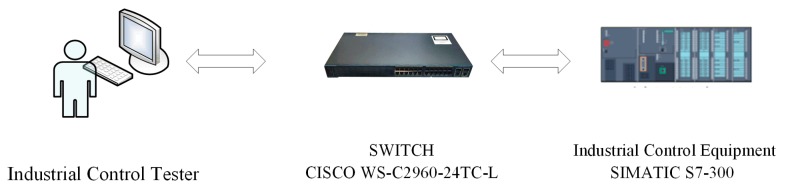
Experimental environment for vulnerability mining of Modbus TCP.

The experimental environment topology of vulnerability mining for Modbus TCP in this paper is shown in Figure 8. Among these, the industrial control equipment was the Siemens water tower and water level ICS; the Siemens PLC model was SIMATIC S7-300, Modbus TCP V2.6 server; the switch was CISCO WS-C2960-24TC-L.The industrial control tester was deployed on a 64 bit Windows 10 system with 8 GB of memory and an Nvidia GTX 1050 Ti GPU. The learning model was based on TensorFlow-GPU 1.8.0, CUDA 9.0, CUDNN 6.0, and Python 3.6.6.

First, all the messages in the dataset with normal responses were filtered out, and then, the repeated messages were removed as the learning data of this study. A total of 40,000 Modbus TCP messages were selected, and 80 epochs were learned. In learning, the learning rate was 0.0001, the parameter τ was 0.6, the model dim was 128 and 256, and the generation model saved every 10 epochs.

### 5.3. Results

Because the generation model described the probability of generating a complete test case, it was not possible to evaluate the learning performance by learning the label of each field accurately. Therefore, this experiment used different epoch learning model parameters to generate test cases, and then, we compared the reception rate of the industrial control equipment and chose the most suitable learning epoch to mine vulnerabilities. In the process of test case generation, the initial sequence of test case generation needed to be changed randomly to ensure that the generated test case met the characteristics of the high coverage rate and high reception rate of the industrial control equipment.

#### 5.3.1. Test Efficiency Analysis

**Figure 9:**
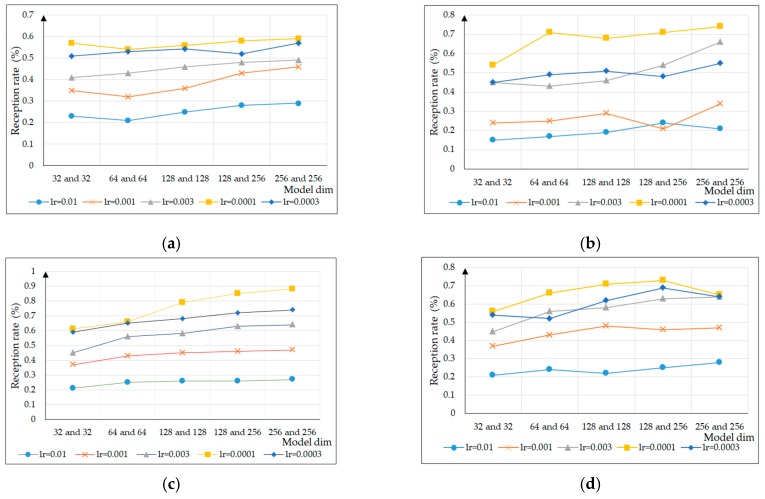
Comparison of super parameter selection in the model under different τ values: (**a**)τ=0.4; (**b**)τ=0.5; (**c**)τ=0.6; (**d**)τ=0.7.

In this experiment, to select appropriate super parameters to maintain the ability of generating test case diversity, firstly, for the parameters, τ was 0.4, 0.5, 0.6, and 0.7, respectively, and the experiment compared the equipment reception rate with learning rates of 0.01, 0.001, 0.003, 0.0001, and 0.0003 and model dim of 32 and 32, 64 and 64, 128 and 128, 128 and 256, and 256 and 256, as shown in Figure 9. With an increase of the parameter τ, the reception rate of test cases generated by different model dim and learning rates for the industrial control equipment was also constantly changing and generally showed a trend of first increasing, then decreasing. When the parameter τ was 0.6, the model dim was 128 and 256, and the learning rate was 0.0001, with the reception rate reaching its maximum.

**Figure 10:**
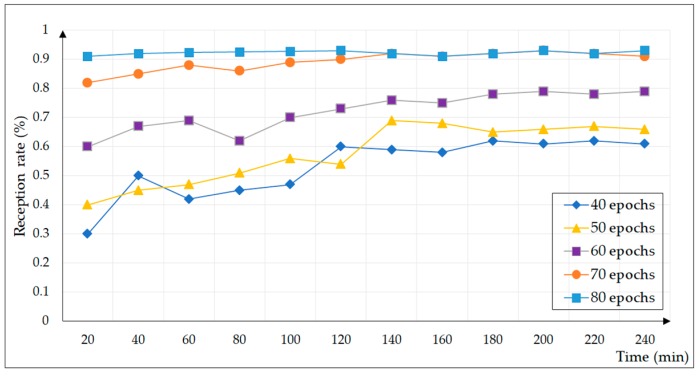
Reception rate over time for learning epochs.

In the experiment, a learning epoch was selected to generate 14400 test cases for 40, 50, 60, 70, and 80, respectively, and a total of four hours of fuzzing test were carried out, as shown in Figure 10. In order to verify the effect of our A-s fuzzer, a test case was sent to the industrial control equipment every second, and the reception rate and vulnerability number of the industrial control equipment were counted every 20 min. The test cases generated by A-s fuzzer were not subjected to any elimination or manual intervention.

As the number of learning epochs increased, the model’s ability to learn message semantics became more accurate. The test cases generated at 40 epochs basically met the requirements of the protocol, and the equipment reception rate increased with time; however, there was a significant difference from the real message. With the continuous increase of the epochs, the reception rate of the industrial control equipment was gradually improved until 80 epochs; approximately 90% of the test cases were received by the experimental equipment. In summary, for the A-s fuzzer test framework, the learning rate was 0.0001 and the model dim was 128 and 256, and it learned 80 epochs to generate test cases for vulnerability mining.

#### 5.3.2. Analysis of the Test Results

In the process of vulnerability mining, each test case had its unique number. During the A-s fuzzer experiment, three types of vulnerability were found, and the case number, abnormal response messages, and vulnerability types are shown in Table 2. Among them, case number represents the test case number when this vulnerability type was first discovered. Each vulnerability was analyzed according to the Modbus TCP document as follows. Vulnerability Numbers 02 and 03 were all emerging Modbus TCP vulnerabilities. All the vulnerabilities were found by A-s fuzzer and are listed and analyzed in Appendix A.

It can be known from Table 2. Vulnerability 01 is the insufficient verification of data authenticity vulnerability. Function code 0x05 was the operation of writing a single coil. In the request Data, the first two bytes represented the coil address to be accessed, which could only be 0xFF00 or 0x0000, and the last two bytes represented the write operation of ON/OFF. In the response Data, the first two bytes were the same as the first two bytes in the request data field, and the last two bytes could only be 0xFF00 or 0x0000, indicating that the write operation was completed. In this test case, the coil with address 0x009B did not judge the parameters submitted by the last two bytes, causing a vulnerability of insufficient verification of data authenticity. 

Vulnerability 02 is the information tampering vulnerability. Function code 0x0F was the operation to write multiple coils. In the request Data, the first two bytes represented the starting address of the coil to be written, the third and fourth bytes represented the number of coils to be written, the fifth byte represented the byte length of the number of coils, and the sixth byte to the end position represented forcing each coil to be ON/OFF. In the response Data, the first four bytes in the request Data were returned, indicating that the coil write operation was completed. In this test case, Data did not force any operation of the coil with address 0x0007–0x0009, but returned that the forced operation was completed, and Modbus TCP was transmitted in clear text. In this way, the attacker could eavesdrop the transmitted coil address and arbitrarily tamper with the forced operation not carried out on the coil, resulting in the vulnerability of information tampering.

Vulnerability 03 is the arbitrary data injection vulnerability. Function code 0x10 was the operation of continuously writing multiple holding registers. In the request Data, the first two bytes represented the start address of the holding register to be written, the third and fourth bytes represented the number of writes, the fifth byte represented the length of the number of bytes, and the sixth byte to the end position represented the value of the data to be written. In the response Data, the first four bytes in the request Data were returned, indicating that multiple write operations of holding registers were completed. In this test case, the data written to the holding register of address 0x0008–0x0009 were not complete, and the low byte data was not written. Therefore, this may lead to the vulnerability of arbitrary data injection.

#### 5.3.3. Comparison of Test Performance

In order to compare the experimental results, the Kitty fuzzer [32], which was applied to the network protocol, and the traditional Peach fuzzer [33] were chosen as the comparison group to generate Modbus TCP test cases. Kitty contains the common functions for each fuzzing process. The industrial control system exploitation framework (ISF) is an industrial control vulnerability utilization framework, which includes an industrial control protocol client and an industrial control protocol module. The fuzzing of the Modbus TCP was carried out by combining the Kitty fuzzing framework with the industrial control protocol component of the ISF framework. Peach fuzzer is a fuzzing framework with an XML format that is easy to understand. It not only has a simple configuration environment, but also can test a variety of data formats. In this study, three types of fuzzers were used to generate Modbus TCP test cases for vulnerability mining. The reception rate of an industrial control equipment, test case variation rate, types of triggering vulnerabilities, the number of test cases used to find the first vulnerability, and vulnerability mining ability were compared and analyzed as follows.

(a) Reception rate (RT): Reception rate refers to the efficiency of test data generation, reflecting the similarity between the generated test case and the real data, as follows:

(21) $$RT=\frac{\mathrm{total}\;\mathrm{number}\;\mathrm{of}\;\mathrm{test}\;\mathrm{cases}\;\mathrm{received}}{\mathrm{total}\;\mathrm{number}\;\mathrm{of}\;\mathrm{test}\;\mathrm{cases}\;\mathrm{sent}}\times 100\%$$

**Figure 11:**
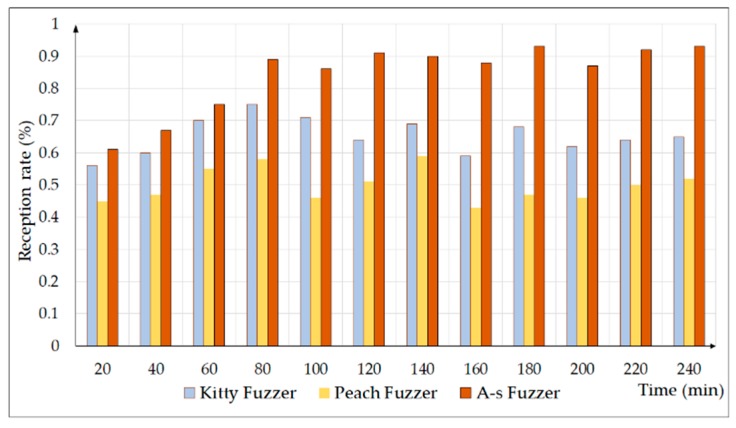
Comparing the reception rate of the three fuzzers. A-s, anti-sample.

Figure 11 shows the reception rate of the test cases generated by the three fuzzers every 20 min. The reception rate of Peach fuzzer was the lowest, and Kitty fuzzer was relatively good. More than half of the test cases could be received by the experimental equipment. The reception rate of A-s fuzzer was the highest, and most of the test cases could be received by the experimental equipment.

(b) Test case variation rate: The second experimental evaluation indicator is the test case variation rate (TCVR), which is defined as the ratio of abnormal test cases in the total test cases generated, as follows:

(22) $$TCVR=\frac{\mathrm{total}\;\mathrm{number}\;\mathrm{of}\;\mathrm{abnormal}\;\mathrm{test}\;\mathrm{cases}}{\mathrm{total}\;\mathrm{number}\;\mathrm{of}\;\mathrm{test}\;\mathrm{cases}\;\mathrm{sent}}\times 100\%$$

This experiment evaluated the *TCVR* of three fuzzers for all the test cases. The specific statistical results are listed in Table 3. In the comparison of the variation rates of all the test cases, A-s fuzzer was slightly lower than Kitty fuzzer, and Kitty fuzzer had the highest *TCVR*, whereas Peach fuzzer had the lowest *TCVR*.

(c) Types of triggering vulnerabilities: Based on the types of vulnerabilities triggered by A-s fuzzer, the number of test cases needed to find the corresponding vulnerabilities by the three methods was counted. The specific experimental results are displayed in Table 4. If there is no fill in the table, that means that no such vulnerability was found. Each fuzzer found each vulnerability in a different order, but the performance of the traditional Peach fuzzer was not good. Although Vulnerability Numbers 01 and 03 could also be found, they were more than the number of test cases required by the other two fuzzers. Kitty fuzzer was the first fuzzer to find the first vulnerability, but in this experiment, there was no test case that could find Vulnerability Number 03. However, the number of test cases needed by A-s fuzzer to find various vulnerabilities was less than the others.

(d) Vulnerability mining ability (VMA): The fourth evaluation indicator was the comparison of the *VMA* of the three methods. *VMA* revealed the average number of test cases required to trigger an exception and calculated not only the number of vulnerabilities found in the test, but also the number of test cases needed to identify these vulnerabilities. The calculation is given by:

(23) $$VMA=\frac{\mathrm{total}\;\mathrm{number}\;\mathrm{of}\;\mathrm{exceptions}\;\mathrm{triggered}}{\mathrm{total}\;\mathrm{number}\;\mathrm{of}\;\mathrm{test}\;\mathrm{cases}\;\mathrm{sent}}\times 100\%$$

As shown in Table 5, the first column compared the time it took for each fuzzer to discover the first vulnerability. The experiment sent one test case per second, which also represented the number of test cases required when the first vulnerability was found. The second column compares the number of vulnerabilities found by each fuzzer in four hours. The third column compares the *VMA* of each fuzzer. As can be seen from the table, the vulnerability mining ability of A-s fuzzer was higher than those of the other fuzzers; the time required to find the first vulnerability was relatively small; and the number of vulnerabilities found within four hours was also the largest. 

## 6. Conclusions

In view of the development of fuzzing and the vulnerability characteristics of the industrial control protocol, this paper designed and implemented a Modbus TCP test case generation model based on the anti-sample idea. 

First, a learning probability distribution based on RNN was proposed to represent the probability distribution relationship between fields in Modbus TCP messages. Specifically, RNN was used to learn the semantics of PDU, and the softmax function was used as the activation function to represent the probability distribution of the field.

Then, in order to construct efficient vulnerability mining test cases, the test case generation method of anti-sample was proposed. According to the probability distribution of the field and the generated random variable threshold, the relationship between the random threshold and the maximum probability in the probability distribution was compared. The result of the comparison determined whether to replace the current data value with the data value of the minimum probability to increase the possibility of industrial control exceptions. According to the protocol specification, the MBAP header was supplemented with random values to form a complete test case.

Finally, a test case generation model based on anti-samples was proposed for the comprehensive probability distribution and anti-sample algorithm. The generation model was divided into the learning stage and the generation stage. The task in the learning stage was to represent the message information of the training dataset through RNN. The task of the generation stage was to use the learned RNN to output the probability distribution corresponding to the data value generated by the current time and to change the case generation strategy according to the anti-sample algorithm. The generated data value was taken as the RNN input field of the next time, so as to generate the complete test case in a cycle.

We successfully captured three types of Modbus TCP vulnerabilities with this experimental method, indicating that A-s fuzzer could effectively detect industrial control protocol vulnerabilities. In the future, the next work is to obtain a more extensive dataset to enhance the persuasion of the generated model and extend A-s fuzzer to apply it to a private protocol.

## 7. Patents

Yingxu Lai, Huijuan Gao, Jing Liu, Wenqian Feng, and Zhidong Wang. Vulnerability Mining Method for the Modbus TCP Protocol Using an Anti-sample Fuzzer. (Application No. or Patent No.: 201910633262.4).

## Figures and Tables

**Table 1:** Abnormal codes and abnormal descriptions.

Abnormal Code	Abnormal Description
01	Non-standard Function Code
02	Illegal Data address
03	Illegal Data value

**Table 2:** Request and response detection results of Modbus TCP exception.

Vulnerability Number	Vulnerability Types	Case Number	Abnormal Response Messages
01	Insufficient Verification of Data Authenticity	3325	request: A1 D3 00 00 00 06 F1 05 00 02 03 0A response: A1 D3 00 00 00 06 F1 05 00 02 03 00
02	Information Tampering	2359	request: 3B B5 00 00 00 07 02 0F 00 07 00 02 01 response: 3B B5 00 00 00 06 02 0F 00 07 00 02
03	Arbitrary Data Injection	2941	request: 0F 5C 00 00 00 08 FB 10 00 08 00 01 02 0F response: 0F 5C 00 00 00 06 FB 10 00 08 00 01

**Table 3:** Statistical table of the variation rate of all the test cases of the three fuzzers. *TCVR*, test case variation rate.

Fuzzer	Number of Normal Test Cases	Number of Abnormal Test Cases	*TCVR* (%)
Kitty Fuzzer	1716	12,684	0.881
Peach Fuzzer	2792	11,608	0.806
A-s Fuzzer	1784	12,616	0.876

**Table 4:** Number of test cases required by the three fuzzers corresponding to various vulnerabilities.

Fuzzer	Vulnerability Number 01	Vulnerability Number 02	Vulnerability Number 03
Kitty Fuzzer	4238	2267	----
Peach Fuzzer	4327	----	5672
A-s Fuzzer	3325	2359	2941

**Table 5:** Comparison of the experimental results of the three fuzzers. *VMA*, vulnerability mining ability.

Fuzzer	Execution Time of the First Vulnerability (s)	Number of Vulnerabilities Found in 4 h	*VMA*
Kitty Fuzzer	2267	13	0.09%
Peach Fuzzer	4327	9	0.06%
A-s Fuzzer	2359	25	0.17%

## Appendix A

In this experiment, all abnormal response messages that cause the insufficient verification of data authenticity vulnerability are shown in Table A1. In the function code 0x05, as long as the last two bytes of the data do not satisfy 0x0000 or 0xFF00, but respond normally, it is considered a vulnerability. The data of these cases do not conform to the data of function code 0x05. They all lead to the vulnerability of insufficient data authenticity verification.

**Table A1:** All the abnormal response messages that cause the insufficient verification of data authenticity vulnerability.

Vulnerability Types	Abnormal Response Messages	
Insufficient Verification of Data Authenticity	request:	A1 D3 00 00 00 06 F1 05 00 02 03 0A
	response:	A1 D3 00 00 00 06 F1 05 00 02 03 00
	request:	9C DE 00 00 00 06 FF 05 00 9B A8 00
	response:	9C DE 00 00 00 06 FF 05 00 9B A8 00
	request:	8A 6C 00 00 00 06 12 05 00 05 02 03
	response:	8A 6C 00 00 00 06 12 05 00 05 02 00
	request:	04 D2 00 00 00 06 58 05 00 0D 02 0E
	response:	04 D2 00 00 00 06 58 05 00 0D 02 00
	request:	10 A2 00 00 00 06 3D 05 00 2C 0F 0A
	response:	10 A2 00 00 00 06 3D 05 00 2C 0F 00
	request:	09 25 00 00 00 06 E2 05 00 4F 00 0C
	response:	09 25 00 00 00 06 E2 05 00 4F 00 00
	request:	15 64 00 00 00 06 A5 05 01 4C 00 1F
	response:	15 64 00 00 00 06 A5 05 01 4C 00 00
	request:	32 18 00 00 00 06 FC 05 01 B5 0D 1F
	response:	32 18 00 00 00 06 FC 05 01 B5 0D 00
	request:	21 D5 00 00 00 06 11 05 00 02 03 1B
	response:	21 D5 00 00 00 06 11 05 00 02 03 00
Insufficient Verification of Data Authenticity	request:	81 45 00 00 00 06 19 05 00 02 05 2B
	response:	81 45 00 00 00 06 19 05 00 02 05 00
	request:	CC 4E 00 00 00 06 3F 05 01 1F 00 0B
	response:	CC 4E 00 00 00 06 3F 05 01 1F 00 00
	request:	A4 6C 00 00 00 06 D2 05 01 EF F0 0B
	response:	A4 6C 00 00 00 06 D2 05 01 EF F0 00
	request:	34 D5 00 00 00 06 E8 05 00 FD 02 0C
	response:	34 D5 00 00 00 06 E8 05 00 FD 02 00
	request:	15 A5 00 00 00 06 AD 05 00 EB 04 1F
	response:	15 A5 00 00 00 06 AD 05 00 EB 04 00
	request:	89 C5 00 00 00 06 92 05 00 C3 04 33
	response:	89 C5 00 00 00 06 92 05 00 C3 04 00
	request:	8B 14 00 00 00 06 85 05 01 00 00 01
	response:	8B 14 00 00 00 06 85 05 01 00 00 00
	request:	3A 28 00 00 00 06 F9 05 00 14 25 FF
	response:	3A 28 00 00 00 06 F9 05 00 14 25 00
	request:	22 DF 00 00 00 06 10 05 00 03 2F 22
	response:	22 DF 00 00 00 06 10 05 00 03 2F 00
	request:	87 41 00 00 00 06 79 05 00 02 25 3B
	response:	87 41 00 00 00 06 79 05 00 02 25 00

In this experiment, all abnormal response messages that cause the information tampering vulnerability are shown in Table A2. As for function code 0x03, it is the operation of read holding registers. In the request Data, the first two bytes represent the address of the holding register to be read, and the last two bytes represent the number of addresses to be read. In the response Data, the first byte represents the number of bytes that have been read. The following bytes represent the value of each holding register. In this test case, the number of registers to be read is missing, but a normal response message is returned. This leads to the information tampering vulnerability.

**Table A2:** All the abnormal response messages that cause the information tampering vulnerability.

Vulnerability Types	Abnormal Response Messages	
Information Tampering	request:	3B B5 00 00 00 07 02 0F 00 07 00 02 01
	response:	3B B5 00 00 00 06 02 0F 00 07 00 02
	request:	2D 8E 00 00 00 03 5D 03 01 01
	response:	2D 8E 00 00 00 07 5D 03 04 00 00 00 00

In the experiment, all abnormal response messages that cause the arbitrary data injection vulnerability are shown in Table A3. As for function code 0x06, it is the operation of writing a single holding register. In the request Data, the first two bytes represent the address of the holding register to be written, and the last two bytes represent the data to be written. The response Data is the same as the request Data field, indicating that the write operation to a single holding register has been completed. In the test cases, in the holding register address 0x03, only the binary value 0x07 is written; in the holding register address 0x01, only the binary value 0x01 is written; and the low byte data are lost, but the response is normal. As for function code 0x03, the register address to be read in this test case is not complete, but returns a normal response. Thus, they have caused the arbitrary data injection vulnerability.

**Table A3:** All the abnormal response messages that cause the arbitrary data injection vulnerability.

Vulnerability Types	Abnormal Response Messages	
Arbitrary Data Injection	request:	0F 5C 00 00 00 08 FB 10 00 08 00 01 02 0F
	response:	0F 5C 00 00 00 06 FB 10 00 08 00 01
	request:	AD 82 00 00 00 05 DE 06 00 03 07
	response:	AD 82 00 00 00 06 DE 06 00 03 07 00
	request:	3B 02 00 00 00 05 3C 06 00 01 01
	response:	3B 02 00 00 00 06 3C 06 00 01 01 00
	request:	2D 8F 00 00 00 03 5F 03 01
	response:	2D 8F 00 00 00 45 5F 03 42 6A FF 00 00 00 00 00 00 00 00 05 05 00 6A 00 00 00 00 00 00 C5 DF 00 00 00 00 00 00 00 00 00 00 00 00 00 00 00 00 00 00 00 00 00 00 00 00 00 00 00 00 00 00 00 00 00 00 00 00 00 00 00 00 00 00 00 00
